# Neutralizing Antibody Responses After SARS-CoV-2 Infection in End-Stage Kidney Disease and Protection Against Reinfection

**DOI:** 10.1016/j.ekir.2021.03.902

**Published:** 2021-04-28

**Authors:** Luke Muir, Aneesa Jaffer, Chloe Rees-Spear, Vignesh Gopalan, Fernando Y. Chang, Raymond Fernando, Gintare Vaitkute, Chloe Roustan, Annachiara Rosa, Christopher Earl, Gayathri K. Rajakaruna, Peter Cherepanov, Alan Salama, Laura E. McCoy, Reza Motallebzadeh

**Affiliations:** 1 UCL Institute of Immunity & Transplantation, University College London, London, UK; 2 UCL Division of Infection & Immunity, University College London, London, UK; 3 Department of Nephrology & Transplantation, Royal Free London NHS Trust, London, UK; 4 Research Department of Surgical Biotechnology, UCL Division of Surgery and Interventional Science, University College London, London, UK; 5 The Francis Crick Institute, London, UK; 6 Centre for Transplantation, Department of Renal Medicine, University College London, London, UK

**Keywords:** ESKD, hemodialysis, antibody, COVID-19, SARS-CoV-2, neutralization assay

## Abstract

**Introduction:**

Patients with end-stage kidney disease (ESKD) represent a vulnerable group with multiple risk factors that are associated with poor outcomes after severe acute respiratory syndrome coronavirus 2 (SARS-CoV-2) infection. Despite established susceptibility to infectious complications and the importance of humoral immunity in protection against SARS-CoV-2, few studies have investigated the humoral immune response to SARS-CoV-2 within this population. Here, we evaluate the seroprevalence of SARS-CoV-2 in patients awaiting renal transplantation and determine whether seroconverted patients with ESKD have durable and functional neutralizing activity against SARS-CoV-2.

**Methods:**

Serum samples were obtained from 164 patients with ESKD by August 2020. Humoral immune responses were evaluated by SARS-CoV-2 spike S1 subunit and nucleoprotein semiquantitative enzyme-linked immunosorbent assay (ELISA) and SARS-CoV-2 spike pseudotype neutralization assay.

**Results:**

All patients with ESKD with reverse-transcriptase polymerase chain reaction (RT-PCR)–confirmed infection (n = 17) except for 1 individual seroconverted against SARS-CoV-2. Overall seroprevalence (anti-S1 and/or anti-N IgG) was 36% and was higher in patients on hemodialysis (44.2%). A total of 35.6% of individuals who seroconverted were asymptomatic. Seroconversion in the absence of a neutralizing antibody (nAb) titer was observed in 12 patients, all of whom were asymptomatic. Repeat measurements at a median of 93 days from baseline sampling revealed that most individuals retained detectable responses although a significant drop in S1, N and nAb titers was observed.

**Conclusion:**

Patients with ESKD, including those who develop asymptomatic disease, routinely seroconvert and produce detectable nAb titers against SARS-CoV-2. Although IgG levels wane over time, the neutralizing antibodies remain detectable in most patients, suggesting some level of protection is likely maintained, particularly in those who originally develop stronger responses.

## Graphical abstract

**Figure:**
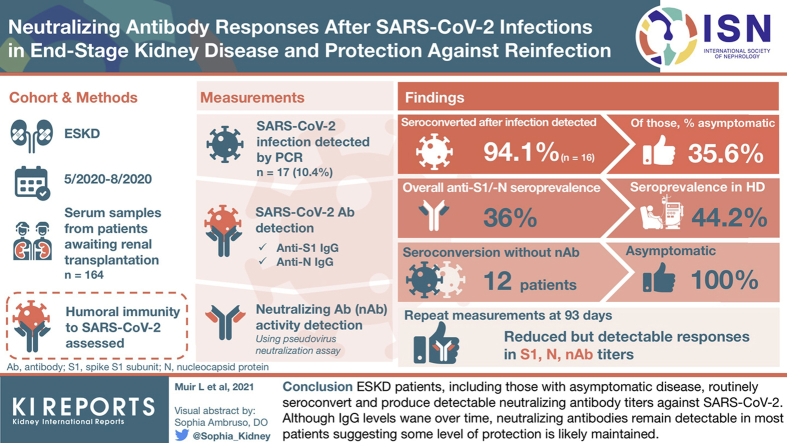

See Commentary on Page 1761

Patients with ESKD represent an extremely vulnerable group with a disproportionate number of recognized risk factors for adverse outcomes after SARS-CoV-2 infection.^1^, ^2^, ^3^ Data from the UK Renal Registry have revealed that by the end of the first wave of the pandemic, 23% of patients receiving incenter hemodialysis (ICHD) and infected with SARS-CoV-2 have died.^4^

Dialysis units are recognized as potential centers for the rapid spread of SARS-CoV-2,^5^^,^^6^ and some of the key questions pertaining to infection in patients with ESKD, in particular those receiving ICHD as they comprise more than 70% of the incident renal transplant population, include quantifying the frequency of asymptomatic infection and determining whether seroconversion is protective against further infection.^7^^,^^8^ Moreover, many of the commercially available assays do not give information on comparable antibody titer, the variety of different antigenic targets that anti–SARS-CoV-2 antibodies (nucleocapsid and spike) are raised to,^9^^,^^10^ or their viral neutralizing ability, which is considered the gold standard for measuring a functional antibody that can inhibit SARS-CoV-2 infection.^11^, ^12^, ^13^, ^14^, ^15^, ^16^ Allied to an overall increased risk of infections, patients with ESKD have impaired cell-mediated and humoral immune responses, leading to lower seroconversion rates and quicker decline of antibody levels as compared with healthy subjects.^17^, ^18^, ^19^, ^20^, ^21^ Whether patients on hemodialysis mount an effective nAb response against SARS-CoV-2 is currently unknown. A more detailed evaluation of the humoral response to SARS-CoV-2 in ESKD is thus required.

Here, we set out to quantify IgG antibody levels to spike S1 subunit (S1) and nucleocapsid (N) proteins of SARS-CoV-2 and evaluate how well these responses correlate with nAb activity.^22^, ^23^, ^24^ Determining the neutralizing ability of SARS-CoV-2 spike antibodies is critical to understanding protection from reinfection^14^^,^^15^^,^^25^ in patients awaiting transplantation and as a consequence likely to receive immunosuppression—a significant risk factor for poor prognosis in SARS-CoV-2 infection.^3^

## Methods

### Patient Selection

A total of 217 patients affiliated with The Royal Free London NHS Trust (London, UK) and, as of 30 May 2020, listed on the National NHS Blood and Transplant waiting list for renal transplantation were included. Clinical and routine pathology data were obtained from electronic and dialysis records. The study was approved by The Royal Free London NHS Foundation Trust—UCL Biobank Ethical Review Committee (RFL B-ERC; reference NC.2018.010). Patients were followed up until 15 January 2021. A total of 57 patients (26 seropositive and 31 seronegative) had received a kidney transplant by this date.

### Data Collection

Demographic information, clinical presentations, chest computed tomography results, laboratory tests, and treatment and outcome data were collected from patient medical records. COVID-19 severity was classified as previously described by Seow *et al*.^26^ Laboratory data collected for each patient included complete blood count, coagulation profile, serum biochemical tests (including renal and liver function, electrolytes, lactate dehydrogenase, and C-reactive protein), serum ferritin, and biomarkers of infection. Relative measures of socioeconomic deprivation were evaluated using the Index of Multiple Deprivation, defined by patient home address postcode using UK government statistics (https://www.gov.uk/government/statistics/english-indices-of-deprivation-2019) and presented as deciles (1 = most advantaged; 10 = most disadvantaged).^27^ Frailty was evaluated using the Rockwood Clinical Frailty Scale (1–2: very fit, well; 3–4: managing well, vulnerable; 5–6: mildly to severely frail; 8–9: very severely frail, terminally ill).^28^^,^^29^

### Diagnosis of COVID-19 Infection

A confirmed diagnosis of COVID-19 was based either oro- or naso-pharyngeal throat swabs for SARS-CoV-2 by RT-PCR after either routine screening or acute presentation.

A confirmed case of COVID-19 was defined as an individual with oro/nasopharyngeal swabs that were positive for SARS-CoV-2 using the laboratory-based PCR test. Symptomatic patients were defined as those with laboratory-confirmed COVID-19 infection with symptoms such as fever, cough, sore throat, and sputum. An asymptomatic case was defined as an individual with a positive PCR test result but without any relevant clinical symptoms in the preceding 14 days or those who were pauci-symptomatic on the basis of the COVID-19 questionnaire survey during the study period and did not undergo a PCR test. Routine asymptomatic swabbing was not performed in the first peak of the pandemic; from October 2020 onward, a weekly SARS-CoV-2 PCR screening practice was instituted for all patients with ICHD.

Patients with a negative IgG antibody assay were considered to be at risk of infection from their first antibody assay to either the end of the study or their first PCR-positive test, whichever occurred earlier. Those with a positive antibody assay result were considered to be at risk of reinfection 60 days after their first positive antibody result to either the end of the follow-up period or their next PCR-positive test, whichever occurred earlier, irrespective of subsequent seroreversion.

### SARS-CoV-2 Antibody Detection

As validated and described previously,^22^^,^^24^^,^^30^ 9 columns of a half-well 96-well MaxiSorp ELISA plate (VWR, Lutterworth, UK) were coated with purified SARS-CoV-2 spike S1 or N protein (Peter Cherepanov, Francis Crick Institute, London, UK) in phosphate-buffered saline (PBS) (Sigma Aldrich, Gillingham, U.K) (3 μg/ml per well in 25 μl) and the remaining 3 columns were coated with 25 μl goat antihuman F(ab)’2 (Sigma Aldrich) diluted 1:1000 in PBS to generate the internal standard curve. After incubation at 4 °C overnight, the ELISA plate was blocked for 1 hour in assay buffer (PBS, 5% milk, 0.05% Tween 20). Sera were diluted in assay buffer in the ratios 1:50 to 1:5000, and 25 μl was added to the ELISA plate. Serial dilutions of known concentrations of IgG standards (Sigma Aldrich) were applied to the 3 standard curve columns in place of the sera. The ELISA plate was then incubated for 2 hours at room temperature and then washed 4 times with PBS and 0.5% Tween 20. Alkaline phosphatase-conjugated goat antihuman IgG (Stratech Scientific, Cambridge, U.K) at a 1:1000 dilution was then added to each well and incubated for 1 hour. The plates were then washed 6 times with PBS and 0.5% Tween 20, and 25 μl of colorimetric alkaline phosphatase substrate (Sigma Aldrich) was added. Absorbance was measured at 405 nm. Antigen-specific IgG serum concentrations were then calculated based on interpolation from the IgG standard results using a 4-parameter logistic regression curve fitted model.

### Neutralization Detection Using Pseudovirus Neutralization Assay

HIV-1 particles pseudotyped with SARS-CoV-2 spike (Wuhan-Hu-1) were produced in a T75 flask seeded the day before with 3 million HEK293T cells (ATCC, Manassas, VA) in 10 ml complete Dulbecco's modified Eagle's medium (Dulbecco's modified Eagle's medium supplemented with 10% fetal bovine serum, 100 IU/ml penicillin, and 100 μg/ml streptomycin) (Sigma Aldrich). Cells were transfected using 60 μg of PEI-Max (Polysciences, Inc., Warrington, PA) with a mixture of the following 3 plasmids: 9.1 μg HIV-1 luciferase reporter vector,^30^ 9.1 μg HIV p8.91 packaging construct, and 1.4 μg WT SARS-CoV-2 spike expression vector.^30^ Supernatants containing pseudotyped virions were harvested 48 hours after transfection, filtered through a 0.45-μm filter, and stored at −80 °C. Neutralization assays were conducted by serial dilution of the serum in complete Dulbecco's modified Eagle's medium and incubated with the pseudotyped virus for 1 hour at 37 °C in 96-well plates. HeLa cells stably expressing angiotensin-converting enzyme 2 (provided by J.E. Voss, Scripps Research Institute, La Jolla, CA) were then added to the assay (10,000 cells per 100 μl per well). After 48 to 72 hours, luminescence was evaluated as a proxy of the infection by lysing the cells with the Bright-Glo luciferase kit (Promega, Madison, WI), using a GloMax plate reader (Promega). Measurements were performed in duplicate and used to calculate 50% inhibitory dilutions (ID50) in GraphPad Prism software (GraphPad, San Diego, CA).

### Statistical Analysis

The 95% confidence interval of seroprevalence was calculated using theb Wilson method. All continuous characteristics are described as either means and SDs or medians and interquartile ranges (IQRs), and categorical characteristics are described as numbers (%). Normally distributed variables were compared using *t* tests, and nonparametric data were compared using the Mann–Whitney *U* test. The Fisher exact tests or chi-square tests were used for proportional assessments. Pairwise correlations were evaluated using nonparametric two-tailed Spearman correlation tests. *P* < 0.05 was considered significant. Statistical analyses were carried out using GraphPad Prism 7.0.

## Results

**Figure 1:**
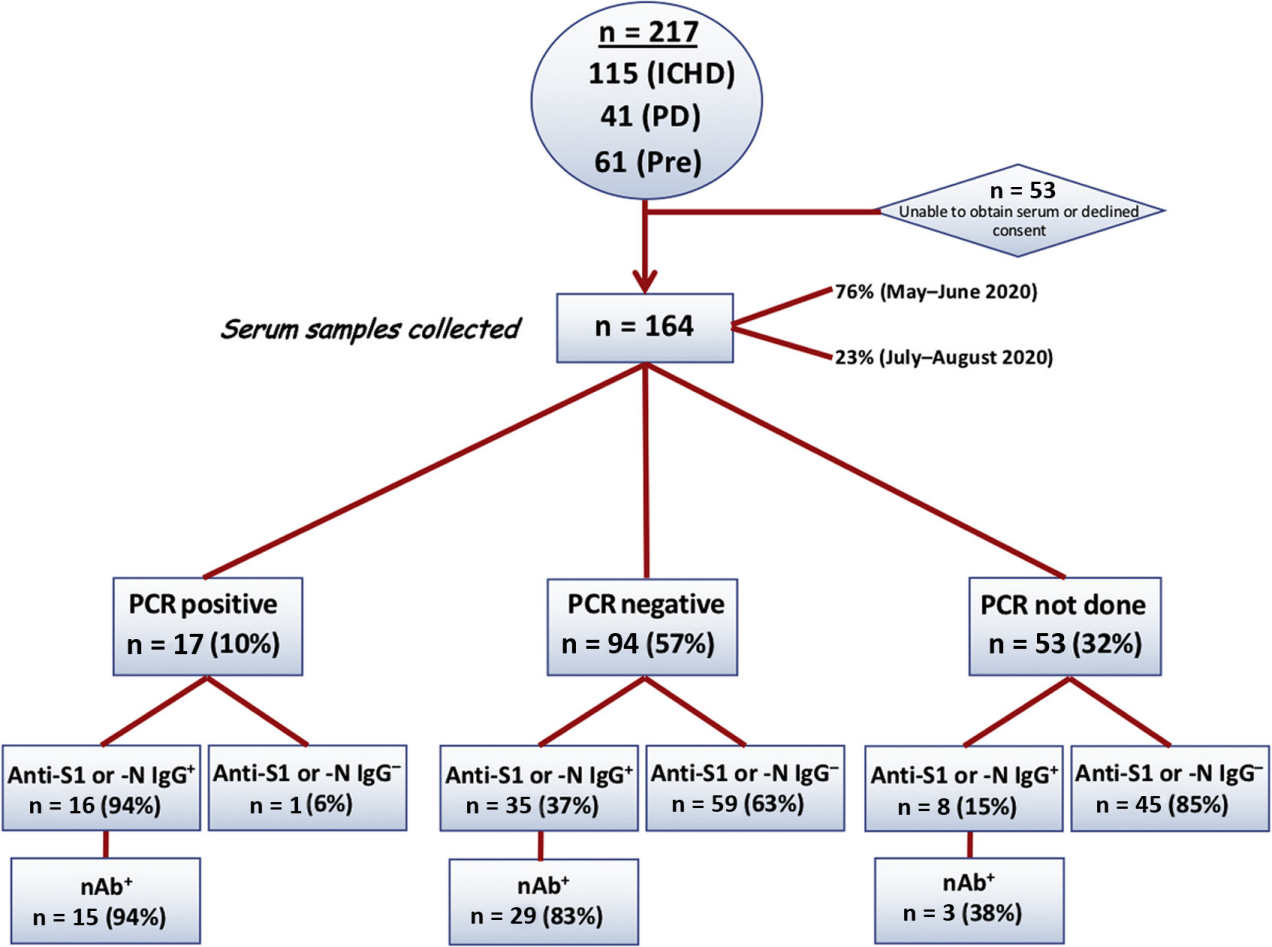
Study flow diagram. A total of 217 patients were eligible for inclusion in the study and 164 were included in the analysis. Flowchart indicates the number of patients in each group by SARS-CoV-2 viral RNA status detected by reverse-transcriptase PCR. ICHD, incenter hemodialysis; N, nucleocapsid; nAb, neutralizing antibody; PCR, polymerase chain reaction; PD, peritoneal dialysis; Pre, predialysis; S1, spike; SARS-CoV-2, severe acute respiratory syndrome coronavirus 2.

We obtained a total of 164 individual serum samples from 217 waitlisted patients; 113 (68.9%) were obtained by June 2020, corresponding to the end of the first peak of infectivity,^4^^,^^31^ and the remainder by August 2020. Of the patients with serum samples, 111 (67.7%) were tested for symptomatic SARS-CoV-2 infection by RT-PCR and 17 individuals were diagnosed with COVID-19, representing 10.4% of the study population (Figure 1).

The overall observed seroprevalence (anti-S1 and/or anti-N IgG) in the population was 36% (n = 59 of 164) and was higher in patients receiving ICHD (44.2%). Seroconversion was not detected in 1 RT-PCR–positive patient (6%). There was no difference in the timing of the tests with a serum sample obtained by June 2020 in 67.8% and 62.9% of SARS-CoV-2 antibody-positive and -negative patients, respectively (*P* = 0.53). Compared with SARS-CoV-2 antibody-negative patients, seroconverted patients were more likely to be from a black, Asian, or minority ethnic background (*P* = 0.08), receive ICHD as opposed to being predialysis or on peritoneal dialysis (*P* = 0.006), have a higher clinical frailty score (*P* = 0.02), and with a significantly fewer proportion on immunosuppression (*P* = 0.001) (Table 1). Indications for immunosuppression and details of the classes of therapy used are found in Table S1.

**Table 1:** Patient characteristics in serologically proven (anti-N and/or anti-S1 SARS-CoV-2 IgG antibody) infection compared with antibody (anti-N and anti-S1)-negative patients

Variables	Antibody positive, n = 59	Antibody negative, n = 105	*P* value
Age (yr), mean (SD)	54.5 (11.9)	53.6 (12.7)	0.67
Male sex, n (%)	40 (67.8)	63 (61.1)	0.43
Ethnicity, n (%)			0.08
BAME	45 (76.3)	65 (61.9)	
Caucasian	14 (23.7)	38 (36.2)	
Index of multiple deprivation decile, median (IQR)	3 (2–5)	4 (3–6)	0.04
Dialysis modality			0.006
ICHD, n (%)	50 (84.7)	63 (60)	
PD, n (%)	4 (6.8)	17 (16.2)	
Predialysis, n (%)	5 (8.5)	24 (22.9)	
Clinical frailty scale, median (IQR)	3 (3-4)	3 (2-3)	0.02
Obesity (body mass index >30 kg/m^2^), n (%)	14 (23.7)	26 (24.8)	0.85
Current or exsmoker, n (%)	18 (23.7)	31 (29.5)	0.97
Cause of ESKD, n (%)			0.003
APKD	6 (10.2)	12 (11.4)	
Diabetic nephropathy	16 (27.1)	13 (12.4)	
Glomerulonephritis	3 (5.1)	25 (23.8)	
Hypertensive	14 (23.7)	11 (10.5)	
Urologic	8 (13.6)	11 (10.5)	
Immunosuppression therapy, n (%)	8 (13.6)	31 (29.5)	0.01
COVID-19 severity classification >1, n (%)	9 (15.3)	3 (2.9)	0.002
Lymphocyte nadir (median), 10^9^/l	0.71 (0.45–1.19)	0.96 (0.51–1.41)	0.09
CRP peak (median), mg/l	47 (14.5–134.8)	14.5 (3–55.5)	0.0007
Ferritin peak (median), mg/l	517 (246.5–891.5)	417 (224–612)	0.05

APKD, autosomal-dominant polycystic kidney disease; BAME, black, Asian, and minority ethnic background; ESKD, end-stage kidney disease; ICHD, incenter hemodialysis; IQR, interquartile range; PD, peritoneal dialysis; SARS-CoV-2, severe acute respiratory syndrome coronavirus 2.

**Figure 2:**
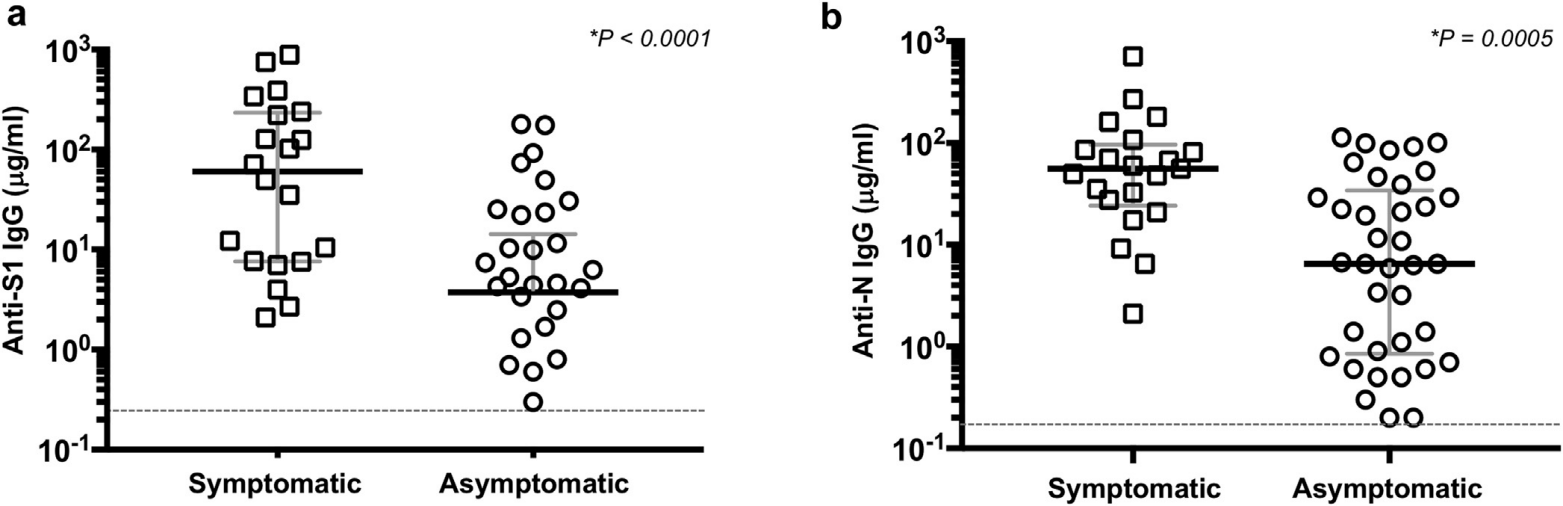
Comparison of virus-specific (a) anti-S1 and (b) anti-N IgG levels (μg/ml) in symptomatic patients (n = 21) and asymptomatic patients (n = 38). The plots reveal medians (black middle line) and first and third quartiles (gray lines). The dashed line indicates the limit of quantification. Comparisons conducted using unpaired, two-sided Mann–Whitney *U* test; *P* values are depicted in the plots.

A total of 21 patients who seroconverted (35.6%) were either asymptomatic with a positive PCR test or were pauci-symptomatic during the study period and did not receive a PCR test. Symptomatic seroconverted patients predominantly had features of cough (30.5%), fever (28.8%), and myalgia (13.6%) on acute presentation, with 10 individuals (13.6%) admitted to hospital. Median titers of both anti-S1 and anti-N IgG were higher in symptomatic compared with asymptomatic patients (S1: 60.3 μg/ml, [IQR = 7.6–234.1] vs. 3.75 μg/ml, [IQR = 0–14.2], *P* < 0.001 and N: 55.9 μg/ml, [IQR = 19.2–96.7] vs. 6.5 μg/ml, [IQR = 0.9–34.1], *P* = 0.0005, respectively; Figure 2), in agreement with previous observations.^22^^,^^26^ Apart from a history of smoking, there were no significant demographic differences between symptomatic and asymptomatic patients (Table 2). Predialysis patients in the seroconverted group had better renal function than equivalent patients who were SARS-CoV-2 antibody negative (median estimated glomerular filtration rate of 16 ml/min [10.5–21.5] vs. 9 ml/min [8–15], *P* = 0.06, respectively). A comparison of antibody titers between patients with seroconverted predialysis and ICHD is found in Table S2.

**Table 2:** Comparison of patient demographics between symptomatic and asymptomatic anti–SARS-CoV-2 N and/or S1 IgG-positive patients

Variables	Symptomatic, n = 21	Asymptomatic, n = 38	*P* value
Age (yr), mean (SD)	54.8 (11.1)	54.4 (12.6)	0.89
Male sex, n (%)	13 (61.9)	26 (68.4)	0.61
Ethnicity, n (%)			0.20
BAME	14 (66.6)	31 (81.5)	
Caucasian	7 (33.3)	7(18.4)	
Index of multiple deprivation decile, median (IQR)	3 (2.75–5)	3 (2–5.5)	0.81
Dialysis modality			0.26
ICHD, n (%)	20 (95.2)	31 (81.6)	
PD, n (%)	1 (4.8)	3 (7.9)	
Predialysis, n (%)	0	4 (10.5)	
Clinical frailty scale, median (IQR)	3 (3–4)	3 (3–4)	0.67
Type I or II diabetes, n (%)	10 (47.6)	14 (36.8)	0.58
Obesity (body mass index >30 kg/m^2^), n (%)	3 (14.3)	11 (52.4)	0.21
Current or exsmoker, n (%)	9 (42.9)	19 (50)	0.60
Immunosuppression therapy, n (%)	3 (14.3)	5 (13.2)	0.90

BAME, black, Asian, and minority ethnic background; ICHD, incenter hemodialysis; IQR, interquartile range; PD, peritoneal dialysis; SARS-CoV-2, severe acute respiratory syndrome coronavirus 2.

**Figure 3:**
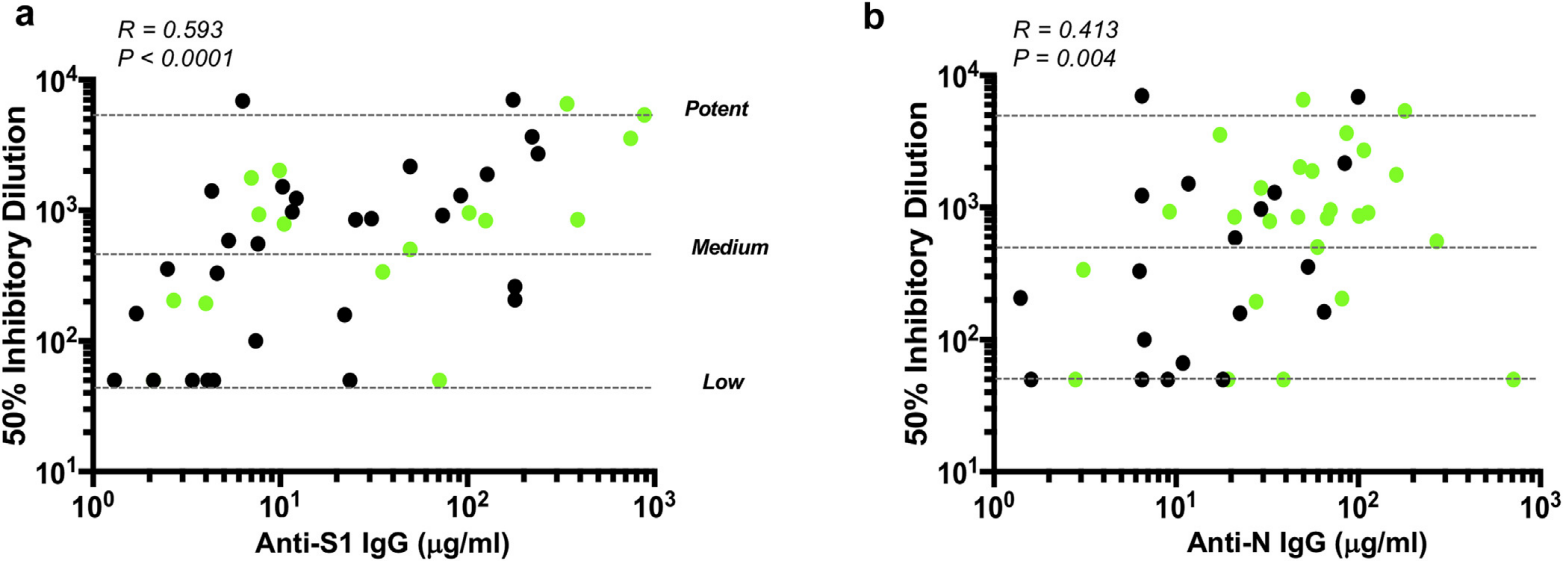
Correlations between S1 and N IgG antibodies with SARS-CoV-2–specific nAbs. Plots of ID50 (y axis) against (a) S1 or (b) N IgG titer (x axis). The dotted line indicates the limit of quantification (ID50 < 50), medium (ID50 < 500), and potent (ID50 > 5000) activities. Sequential serum samples from seropositive patients were titrated in duplicate and preincubated with luciferase-encoding HIV pseudotyped with the SARS-CoV-2 spike for 1 hour before the addition of HeLa cells expressing human ACE2. The *R* and *P* values for the correlations in a and b were determined by two-tailed Spearman’s test. Asymptomatic and symptomatic individuals are revealed in black and green, respectively. ACE2, angiotensin-converting enzyme 2; ID50, 50% inhibitory dilutions; nAb, neutralizing antibody; SARS-CoV-2, severe acute respiratory syndrome coronavirus 2.

**Figure 4:**
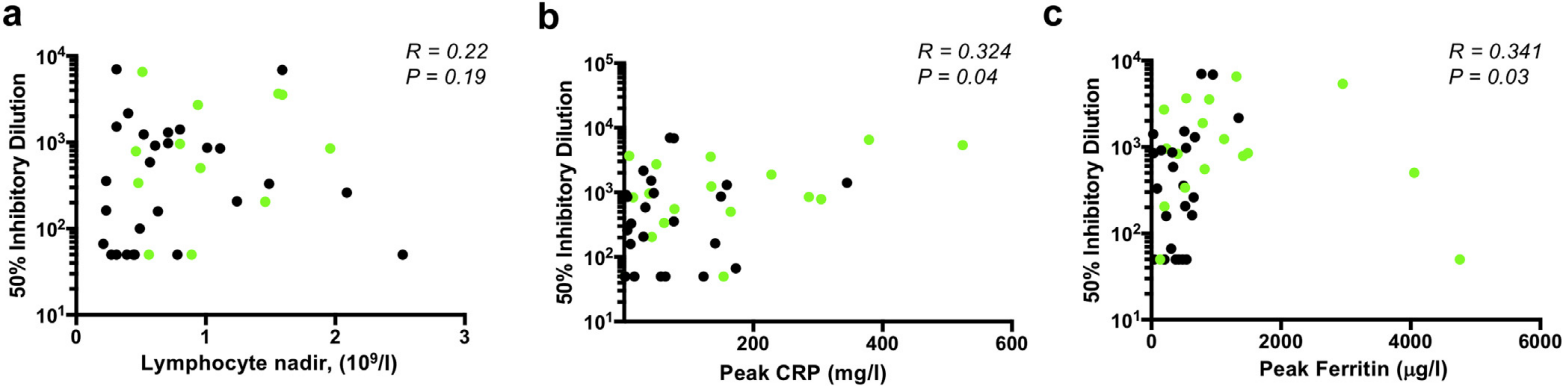
Biochemical and hematological correlates of SARS-CoV-2–specific nAb titers. Correlation of nAb titers with nadir lymphocyte, peak CRP, and peak ferritin levels either at time of positive PCR testing or at time of serum sampling if no or negative PCR test. The *R* and *P* values for the correlations were determined by two-tailed Spearman’s test. Asymptomatic and symptomatic individuals are revealed in black and green, respectively. nAb, neutralizing antibody; PCR, polymerase chain reaction; SARS-CoV-2, severe acute respiratory syndrome coronavirus 2.

The S1 subunit contains the receptor-binding domain, which mediates viral binding to angiotensin-converting enzyme 2 (ACE2) receptors on susceptible cells and is the main target for SARS-CoV-2 nAbs.^32^ We tested the neutralizing activity using a luciferase-encoding–attenuated HIV-1 pseudotyped with the spike protein. A total of 12 patients (20.3%) had low (ID50: 50–200), 7 (11.9%) medium (ID50: 201–500), 17 (28.8%) high (ID50: 501–2000), and 11 (18.6%) potent (ID50: >2001) neutralizing titers. Levels of anti–S1- and -N IgG antibodies correlated strongly with ID50 (Figure 3a and b). Absence of neutralizing activity (ID50 < 50) was found in 12 seroconverted patients (20.3%), which was consistent with their lower anti-S1 and anti-N levels compared with patients who had detectable neutralizing activity (anti-S1 IgG: 0.6 μg/ml, [IQR = 0.53–0.6] vs. 10.5 μg/ml, [IQR = 4.1–92.1], *P* < 0.0001, and anti-N IgG: 0.9 μg/ml, [IQR = 0.5–1.4] vs. 29.2 [IQR = 9–70.3], *P* < 0.0001, respectively). All seropositive patients with no neutralizing activity had asymptomatic infection and were slightly older than those with detectable nAb (median age: 60 years, [IQR = 52.8–75] vs. 54.7 [IQR = 45.2–63.1], *P* = 0.37, respectively). Accordingly, symptomatic patients had higher titers of nAbs, with levels strongly correlated with peak serum ferritin and CRP levels (Figure 4).

**Figure 5:**
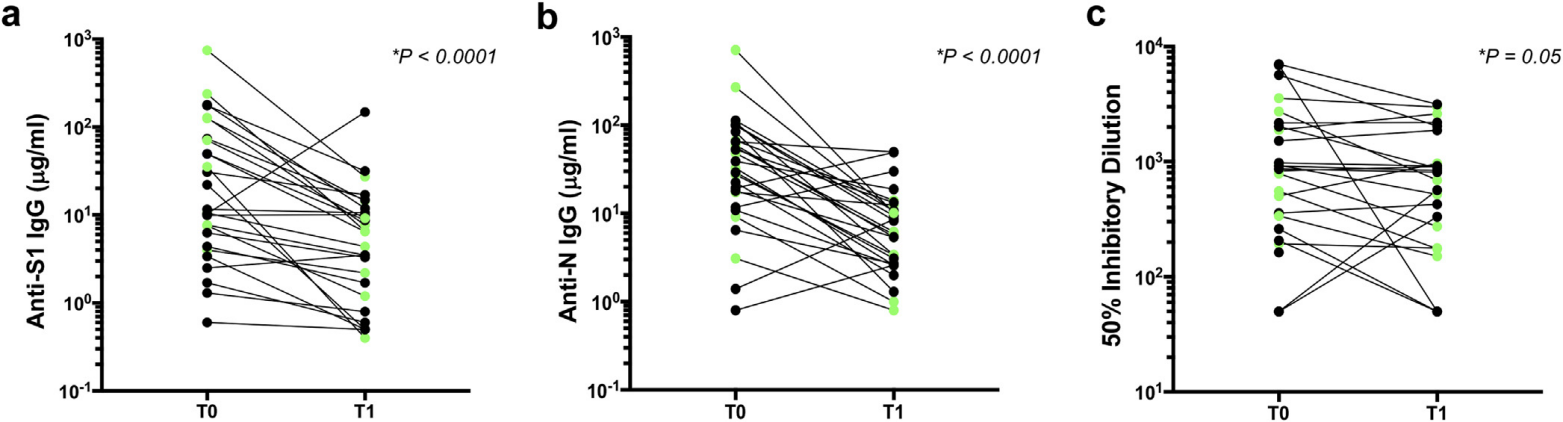
Changes in virus-specific IgG levels and nAb titers of seropositive patients. Each line represents 1 individual. T0 and T1 taken at a median of 92.5 days (IQR = 70.8–111) for anti-S IgG (n = 27), at 92 days (IQR = 69.5–111) for anti-N IgG (n = 28), and at 93 days (IQR = 73–111) for neutralizing antibodies (n = 26). Asymptomatic and symptomatic individuals are revealed in black and green, respectively. Statistical significance was determined using Wilcoxon-matched pair signed-rank test. IQR, interquartile range; N, nucleocapsid; nAb, neutralizing antibody; T0, baseline; T1, repeat samples.

Where possible, we obtained repeat sera and measured S1, N, and neutralizing antibodies from seroconverted patients at a median of 93 days from baseline sampling. There were significant reductions in anti-S1 IgG (*P* < 0.0001), anti-N1 IgG (*P* < 0.0001), and nAb titers (*P* = 0.05) (Figure 5). Of the seroconverted cases with samples at least 40 days from baseline, 2 of the 30 patients seroreverted for anti-S1 IgG and lost nAb activity; both had an initial weak nAb titer (ID50 < 200) and had been transplanted in the intervening period.

Clinical data were obtained from patients at a median of 195 days (IQR = 123–217) after baseline negative anti-S1 and -N IgG and for 202 days at risk (IQR = 109.3–216) after a positive S1 and/or N IgG. There were 3 deaths in the seronegative group; none were related to COVID-19. Of the 105 seronegative patients, 12 subsequently had a positive PCR result (8.58 per 10,000 days at risk), 7 during asymptomatic screening and 5 while being symptomatic. Of the 59 seropositive patients at baseline, one had a positive PCR test 202 days later (overall = 1.26 per 10,000 days at risk); the patient was asymptomatic and had weak baseline (ID50–194) and repeat (ID50–177 at 110 days) neutralizing activities. The incidence rate ratio for positive PCR tests in seroconverted patients was 0.15 (95% confidence interval, 0.003–0.98, *P* = 0.04).

## Discussion

Only a few studies, predominantly conducted with commercial serologic assays, have investigated SARS-CoV-2 prevalence in patients on maintenance dialysis,^33^, ^34^, ^35^, ^36^, ^37^ with some of them having failure of seroconversion after documented COVID-19 infection^33^^,^^38^ which could be related to the lower sensitivity of the assays used.^39^^,^^40^ Although S1 and receptor-binding domain antibodies can provide information on functional immunity given reported correlations with neutralizing activity,^16^^,^^26^^,^^41^^,^^42^ this has not yet been found for patients on dialysis. To address this, we used an inhouse high-throughput serum neutralization assay directed at the spike protein that is well correlated both with inhibition of infection in live SARS-CoV-2 assays^43^, ^44^, ^45^ and with ID50 titers that are associated with protective immunity against secondary infection.^15^^,^^46^, ^47^, ^48^, ^49^

We found that patients with ESKD routinely seroconverted and produced neutralizing antibodies after SARS-CoV-2 infection, including a large number of individuals who were asymptomatic and receiving maintenance hemodialysis between 2 and 3 times per week in the high-exposure setting of dialysis facilities, where maintenance of effective social distancing from other patients or health care workers is logistically challenging. Although titers of the IgG antibody in patients on dialysis decline significantly by 90 days, there is still detectable neutralizing activity, in keeping with reports in non-ESKD individuals that primary infection can provide up to 85% protection against reinfection for at least 6 months.^16^^,^^26^ The only cases in which there was reduction in neutralizing activity to below the threshold of detection included 2 patients who had been transplanted and were receiving maintenance immunosuppression.

Patients who developed antibody responses to SARS-CoV-2 had higher clinical frailty scores, which would be in keeping with previous work revealing that increased frailty is associated with the severity of COVID-19 and durable SARS-CoV-2 spike antibody responses.^3^^,^^50^, ^51^, ^52^ Nevertheless, this is also a reflection of the very high proportion of seroconverted patients comprised those with ICHD, a population that is well-known to be at higher risk of accelerated aging and frailty.^53^^,^^54^ Accordingly, we found a lower, albeit nonsignificant, titer of antibodies in predialysis patients, a group who are able to shield more effectively and thus would likely have had a lower number of exposures compared with patients with ICHD, in which attendance of regular dialysis sessions in a hospitalized setting is associated with a higher risk of nosocomial acquisition of SARS-CoV-2 infection.^6^^,^^55^, ^56^, ^57^ Our dialysis centers have since used numerous strategies, such as changing nursing practice, reducing the number of patients in waiting rooms, limiting shared patient transport, and carrying out regular PCR screening, to minimize the risk of COVID-19 transmission.^58^^,^^59^

Infections are the second leading cause of death among dialysis-dependent patients with ESKD mainly owing to the impairment of both innate and acquired immunities, related to both uremia and concomitant immunosuppression therapy.^60^ Specific uremia-related disturbances in acquired responses include reduced expression of costimulatory molecules on CD4+ T cells and impaired proliferative responses.^61^, ^62^, ^63^, ^64^ Moreover, our previous work has revealed that changes in adaptive immunity in ESKD can be identified before transplantation, such as alterations in the cytokine profiles of regulatory B cells, which are associated with subsequent likelihood of allograft rejection.^65^, ^66^, ^67^ Furthermore, immunosuppression therapy for management of autoimmune renal disease or prevention of human leukocyte antigen sensitization in patients with previously failed transplants,^68^ (the latter was common in our cohort) has effects on humoral, cell-mediated immunity and neutrophil function.^69^^,^^70^ As cognate CD4+ T cell help is critical for the differentiation of antigen-specific B cells (by extrafollicular responses or germinal center reactions) into memory B cells, antibody-secreting plasmablasts, and plasma cells,^71^, ^72^, ^73^, ^74^, ^75^, ^76^, ^77^, ^78^, ^79^, ^80^ the deleterious consequences of B cell lymphopenia in ESKD^81^ are compounded by impaired T-cell–dependent activity of B cells and are reflected in poor serologic responses to T-cell–dependent vaccines.^63^^,^^82^, ^83^, ^84^ Accordingly, although only 1 patient failed to seroconvert after PCR-confirmed SARS-CoV-2 infection, in keeping with the test sensitivity of 92.1%,^24^ we found a higher proportion of patients on maintenance immunosuppression in the group that did not seroconvert. The latter patients were also receiving more intense therapy, in particular, a triple combination of steroid, antimetabolite, and calcineurin inhibitors, compared with their seroconverted counterparts. It is possible therefore that the durability of anti–SARS-CoV-2 antibody responses, especially in response to mild infections, is poorly sustained on a background of immunosuppression therapy and might have accounted for the lack of detectable antibody in some patients by the time serum samples were acquired after the first peak of the pandemic. In support, the 2 patients in our cohort who lost neutralizing activity after 40 days, had initial weak titers and had been transplanted in the intervening period, having received induction immunosuppression with basiliximab and having been maintained on triple therapy. Of note, up to one-third of renal transplant recipients fail to seroconvert after RT-PCR–confirmed SARS-CoV-2 infection^35^^,^^85^ and thus point to the major role of immunosuppression abrogating protective anti–SARS-CoV-2 antibody responses.

With the accelerated spread of new variants (B.1.1.7, B.1.351, and P.1) containing mutations in the spike protein, concerns have been raised on the ability of humoral responses induced by the original Wuhan-Hu-1 strain to neutralize these variants.^86^, ^87^, ^88^ Recent analysis of serum neutralization of the UK variant B.1.1.7 has revealed comparable titers for samples from both mild and severe diseases, with a substantial decrease in the titer (3–10 fold) in <10% of individuals.^89^^,^^90^ Nevertheless, despite this drop in titer in a few cases, neutralization against B.1.1.7 pseudotypes remains detectable, and so it seems likely that the antibody levels observed in this study would also protect from B.1.1.7 infection, although without a numerically defined correlation of protection, this is not absolute. In contrast, most serum samples tested to date^91^^,^^92^ have lost all activity against the South African variant B.1.351.^91^ Therefore, it is likely that individuals would similarly have reduced activity against B.1.351 in the absence of a vaccine boost.^93^^,^^94^

The limitations of our study include the use of a SARS-CoV-2 spike pseudotype neutralization assay to determine neutralization titer rather than a live virus neutralization assay. Nevertheless, previous work has revealed that there is little difference in the neutralization titers determined by the live virus and pseudotyped neutralization assays, suggesting both assays allow for informative determination of the SARS-CoV-2 serum neutralization titer.^44^ Although the correlates of protection from SARS-CoV-2 have not been definitively identified, we know that a neutralization titer as low as 1:50 determined by the pseudotype neutralization assay has been linked to protection in nonhuman primate studies.^46^^,^^49^ Furthermore, the results of vaccination trials to date (using spike-only antibody-targeted vaccines) strongly suggest that antispike antibodies play a major role in protection from infection, which is consistent with the results for many other viruses. Therefore, we are confident that the neutralization titer determined in this study will be linked to some form of protection from SARS-CoV-2. Nevertheless, for the general population, only longitudinal reinfection studies will be able to confirm this. A further limitation is that our spike-reactive IgG titers were evaluated using the spike S1 subunit and not the whole spike protein. This is largely owing to the inherent instability of the native spike protein and the presence of cross-reactive epitopes to seasonal coronaviruses within other spike subunits. The S1 ELISA has been extensively validated and found to be highly specific when tested against >200 prepandemic controls including >100 samples from recent viral/bacterial infections and, importantly, revealed to have no reactivity when tested against 16 seasonal coronavirus infection samples.^24^ One final limitation of the study is the heterogeneity in the PCR testing of patients for SARS-CoV-2 infection. Although screening at ICHD units became a regular practice after the first peak of the pandemic, it is possible that we missed infections in asymptomatic patients during this phase, especially if they were either on predialysis or peritoneal dialysis. Therefore, there might be a higher proportion of patients with PCR-confirmed SARS-CoV-2 infection who failed to seroconvert that we did not detect. Nevertheless, the overall seroprevalence (anti-S1 and/or anti-N IgG) of 44% in our ICHD cohort is almost double the rate of laboratory-confirmed (by positive SARS-CoV-2 swab) COVID-19 cases in London reported to the UK Renal Association,^95^ confirming that asymptomatic disease in patients on dialysis is common and highlights the limitations of early diagnostic screening strategies. We have been using a weekly swab test of all patients in our dialysis centers since the latter half of last year.

Our findings could be applied in risk stratifying patients on dialysis awaiting transplantation and have important implications when considering the potential durability of protection from reinfection.^38^ As the rollout of COVID-19 vaccination programs has commenced in many countries, our results can be used for evaluating the comparative magnitude, durability, and degree of protection of patients on dialysis, a group not represented in any vaccination studies thus far.^96^^,^^97^ Teleologically, waitlisted patients with ESKD who have mounted a robust nAb response to SARS-CoV-2, whether as a result of primary infection or vaccination, might be better protected against reinfection if and when transplanted. Serologic monitoring with neutralizing activity after vaccination will be critical for these patients and is the subject of current work.

## Disclosure

All the authors declared no competing interests.

## Supplementary Material

Supplementary File (PDF)

**Table S1.** Details of immunosuppression therapy.

**Table S2.** Comparison of SARS-CoV-2 IgG and neutralization titers between patients on predialysis and maintenance ICHD.

**STROBE Statement** (PDF).
