# Supplementary material for: Neutralizing Antibody Responses After SARS-CoV-2 Infection in End-Stage Kidney Disease and Protection Against Reinfection
Source: Kidney Int Rep. 2021 Apr 28;6(7):1799–809. doi: 10.1016/j.ekir.2021.03.902 (PMC8081267; doi:10.1016/j.ekir.2021.03.902)
Supplement: Supplementary File (PDF) [file mmc1.pdf]

Table S1. Details of immunosuppression therapy.

Variable	Seroconverted & receiving IS, n=8	Not seroconverted & receiving IS, n=31	p value
Indication for immunosuppression therapy, n (%)			0.40
Previous Transplant	6 (75)	21 (67.7)	
IgA Nephropathy	1 (12.5)	1 (3.2)	
FSGS	1 (12.5)	0	
Microscopic Polyangiitis	0	1 (3.2)	
Lupus nephritis	0	4 (12.9)	
GN	0	2 (6.5)	
MPGN	0	1 (3.2)	
Amyloid	0	1 (3.2)	
Type of immunosuppression, n			0.05
Prednisolone + MMF/Aza + CNI	0	10	
Prednisolone + MMF/Aza	1	4	
Prednisolone + CNI	0	4	
MMF/Aza + CNI	0	2	
Prednisolone	2	5	
MMF/Aza	1	1	
CNI	4	2	
Anti-TNF	0	3	

IS, immunosuppression; FSGS, focal segmental glomerulosclerosis; GN, glomerulonephritis; MPGN, membranoproliferative glomerulonephritis; MMF, Mycophenolate mofetil; Aza, Azathioprine; CNI, calcineurin inhibitor.

Table S2. Comparison of SARS-CoV-2 IgG and neutralization titers between predialysis and maintenance ICHD patients.

	**Pre-dialysis (n=5)**	**ICHD (n=50)**	p value
Titre of anti-S1 antibody, μg/ml median (IQR)	4.1 (0-8.65)	7.6 (1.6-78.2)	0.16
Titre of anti-N antibody, μg/ml median (IQR)	6.5 (0.7-27.4)	22.4 (3.1-67.4)	0.14
ID50, median (IQR)	50 (0-1062)	528.5 (50-1580)	0.12
